# Development of a consensus-based core outcome set for post-treatment recovery in adults with epilepsy and comorbid depression or anxiety: A Delphi and ICF-guided protocol

**DOI:** 10.1371/journal.pone.0330617

**Published:** 2025-08-22

**Authors:** Yan Wang, Zaibang Feng, Zhiyue Guo, Hongxia Xing, Zhaohong Guo, Xinli Zhao, Zubing Mei

**Affiliations:** 1 Department of Neurology, The First Affiliated Hospital of Xinxiang Medical University, Xinxiang, China; 2 Department of Rehabilitation Medicine and Physiotherapy, Changhai Hospital, Naval Military Medical University, Shanghai, China; 3 Department of Physiology and Pathophysiology, School of Basic Medical Sciences, Xinxiang Medical University, Xinxiang, China; 4 School of Health Management, Xinxiang Medical College, Xinxiang, China; 5 Department of Neurosurgery, Zhanjiang Central People’s Hospital, Zhanjiang, Guangdong Province, China; 6 Department of Neurosurgery, The First Affiliated Hospital of Xinxiang Medical University, Zhengzhou, Henan Province, China; 7 Department of Anorectal Surgery, Shuguang Hospital, Shanghai University of Traditional Chinese Medicine, Shanghai, China; 8 Anorectal Disease Institute of Shuguang Hospital, Shanghai, China; University of Alabama at Birmingham, UNITED STATES OF AMERICA

## Abstract

**Introduction:**

Up to 50% of adult patients with epilepsy experience comorbid depression or anxiety, complicating post-treatment recovery and requiring tailored outcome assessment. No core outcome set (COS) exists for this dual-diagnosis population, unlike broader epilepsy COSs or quality-of-life COSs for drug-resistant epilepsy, which do not prioritize mental health comorbidities. This protocol outlines the development of a standardized COS to evaluate post-treatment recovery in adults with epilepsy and comorbid depression or anxiety, using the International Classification of Functioning, Disability, and Health (ICF) framework to ensure a biopsychosocial perspective. This COS aims to enhance cross-study comparability and inform personalized care for this underserved group.

**Methods and analysis:**

This study will employ a three-phase process: (1) A systematic review of outcomes in epilepsy with comorbid depression or anxiety in adult patients, searching PubMed, Embase, and Cochrane Library per Preferred Reporting Items for Systematic Reviews and Meta-Analyses (PRISMA) guidelines, including outcomes such as seizure control, mood stability, cognitive function, social reintegration, and suicide-related outcomes; (2) Mapping outcomes to ICF domains (body functions, activities, participation) for comprehensive coverage; and (3) A three-round Delphi survey using a 9-point Likert scale to achieve consensus among an international panel of ≥60 stakeholders, including neurologists, psychiatrists, psychologists, patients, and caregivers, with ≥20% from low- and middle-income countries. Consensus requires ≥70% agreement on scores of 7–9 (critically important) and ≤15% on 1–3 (low importance). The COS will capture domains like seizure control, mood stability, cognitive function, and social reintegration.

**Discussion:**

This COS will address a critical gap by standardizing outcome measurement for adults with epilepsy and mental health comorbidities, complementing broader COSs, such as the Epilepsy Outcome Set for Effectiveness Trials (EPSET), by focusing on mental health recovery, including suicide-related outcomes, and extending quality-of-life-focused sets, such as those using the Quality of Life in Epilepsy (QOLIE) scales, to include neurological and functional outcomes. The ICF-guided, patient-inclusive approach ensures global relevance. Potential limitations, including stakeholder bias, will be mitigated through diverse recruitment and robust consensus methods.

**Conclusion:**

This protocol leverages Delphi methodology and ICF principles to develop a novel COS for a complex dual-diagnosis population. By prioritizing mental health recovery in adults, including critical outcomes like suicide prevention, it offers a distinct tool to enhance research and clinical practice, with future validation planned to ensure applicability across settings.

**PROSPERO registration number:**

CRD42024576141

## Introduction

Epilepsy is one of the most prevalent neurological disorders, affecting approximately 50 million people globally [1]. This condition is characterized by recurrent, unprovoked seizures resulting from abnormal electrical activity in the brain. Beyond the significant burden of seizure-related symptoms, many adult patients with epilepsy experience various mental health and psychosocial challenges, further complicating their treatment and prognosis [2,3]. Among these, comorbid mental health disorders—particularly depression and anxiety—are highly prevalent and contribute considerably to the overall disease burden [4]. It is estimated that up to 30–50% of adult patients with epilepsy also suffer from major depressive disorder or anxiety disorders, which are known to exacerbate the impact of epilepsy on patients’ quality of life, social functioning, and treatment adherence [5,6]. Such comorbid conditions often lead to poorer outcomes, increased healthcare utilization, and elevated risks of suicidal ideation, suicide attempts, and completed suicide [7].

The impact of depression and anxiety on adult patients with epilepsy is profound and multifaceted. Depression, for example, has been associated with higher rates of suicidal ideation and suicide attempts in epilepsy populations [8,9], highlighting the need for comprehensive, proactive mental health management and suicide prevention strategies in these patients. Anxiety disorders, on the other hand, can increase seizure frequency due to heightened physiological arousal, further impairing patients’ ability to manage their condition effectively [10]. Moreover, comorbid mental health disorders are known to interfere with treatment adherence [11,12], as patients with anxiety or depression may exhibit lower motivation to adhere to prescribed antiepileptic medications or lifestyle modifications.

Post-treatment recovery, defined as the multidimensional process of improving or stabilizing clinical, functional, and psychosocial outcomes following interventions for epilepsy and comorbid depression or anxiety in adults, includes reductions in seizure frequency, improvement or stabilization of mood and anxiety symptoms, enhanced quality of life, and better social and functional participation, and reduction in suicide risk, without implying complete resolution of epilepsy or mental health conditions. The term emphasizes ongoing management through interventions such as antiepileptic drugs, antidepressants, psychotherapy, neuromodulation, or behavioral therapies. Despite these challenges, no standardized core outcome set (COS) exists to assess post-treatment recovery in adults with epilepsy and comorbid depression or anxiety, hindering tailored care and research synthesis. A COS is a standardized, consensus-based set of outcomes that should be measured and reported in all clinical trials and practice for a specific condition to ensure consistency and comparability [23,24]. For example, the International Consortium for Health Outcomes Measurement (ICHOM) developed a COS for chronic kidney disease (CKD), which includes outcomes such as survival (date and cause of death), burden of disease (hospital admissions and days in hospital), cardiovascular events (e.g., myocardial infarction, stroke), and patient-reported health-related quality of life (HRQoL) assessed using tools like PROMIS Global Health, RAND-36, or EQ-5D-5L [35]. These outcomes were selected through a systematic literature review, registry reviews, and a modified Delphi process involving a diverse working group of clinicians, researchers, and patient representatives from nine countries. The outcomes are mapped to the International Classification of Functioning, Disability, and Health (ICF) domains, covering physical function, mental health, and social roles, ensuring a holistic evaluation of CKD patients’ health. Treatment-specific outcomes, such as kidney function (estimated glomerular filtration rate) and albuminuria, are also included for patients with CKD or kidney transplantation [35]. Similarly, our COS will aim to include clinical outcomes, patient-reported outcomes, suicide-related outcomes and functional outcomes, ensuring a comprehensive evaluation of recovery in adult patients across relevant interventions like antiepileptic drugs, antidepressants, psychotherapy, and neuromodulation.

Current assessment approaches for post-treatment recovery in epilepsy often rely heavily on self-reported clinical symptoms and subjective evaluations by healthcare providers [13]. Existing COSs, such as the Epilepsy Outcome Set for Effectiveness Trials (EPSET) for adult epilepsy clinical trials, focus primarily on seizure control, adverse events, and general health outcomes, with limited emphasis on mental health recovery or suicide risk [14]. Similarly, a COS for quality of life in drug-resistant epilepsy prioritizes QOL domains but does not integrate neurological and mental health outcomes relevant to depression or anxiety or suicide [15]. Tools like the Quality of Life in Epilepsy (QOLIE) scales capture broad psychosocial impacts but lack specificity for mental health comorbidities and suicide-related outcomes, limiting their utility for this dual-diagnosis population [16]. This gap leaves clinicians and researchers without a comprehensive framework to evaluate the interplay of seizure control, mood stability, cognitive function, social reintegration, and suicide prevention—key dimensions for adult patients with these comorbidities.

The development of a core outcome measure set offers a solution to these challenges by establishing standardized benchmarks that can be used across clinical trials and routine practice. The ICF framework, developed by the World Health Organization, provides a comprehensive model for understanding health and disability [17,18]. The ICF framework captures the interplay of biological, psychological, and social factors, making it an ideal foundation for developing a core outcome set that considers the multidimensional aspects of health [19,20]. By mapping recovery outcomes onto ICF domains—such as physical health, mental health, suicide risk, and social participation—researchers can create a more holistic tool that is better suited to the needs of adult patients with comorbid mental health disorders. The adoption of an ICF-based outcome set in epilepsy research could enhance the ability to monitor recovery across diverse patient populations and settings, ultimately leading to improved treatment strategies and better long-term outcomes [21,22].

This study addresses the following research questions: (1) What are the key outcomes for assessing post-treatment recovery in adults with epilepsy and comorbid depression or anxiety, including suicide-related outcomes, as identified through a systematic review and stakeholder consensus? (2) How can these outcomes be mapped to the ICF framework to ensure comprehensive coverage of neurological, psychological, suicide-related, and social domains? (3) Which outcomes achieve international stakeholder consensus as essential for inclusion in a COS for this population? This study protocol outlines the development of an internationally agreed-upon core outcome measure set for evaluating post-treatment recovery in adult patients with epilepsy and co-occurring depression or anxiety disorders. To achieve this, we will employ a Delphi methodology, a structured, iterative process that enables the systematic synthesis of expert opinion to reach consensus. The anticipated outcome is a validated, consensus-based COS that complements existing sets by focusing on mental health recovery, including suicide prevention, enabling clinicians and researchers to capture a comprehensive view of recovery beyond seizure control. This standardized outcome set is expected to facilitate cross-study comparisons, support evidence synthesis in systematic reviews, and guide the development of personalized treatment approaches. The COS will apply to all relevant interventions for this population, including antiepileptic drugs, antidepressants, psychotherapy, neuromodulation, and behavioral therapies, ensuring comprehensive outcome assessment across treatment modalities. Furthermore, it will provide a valuable resource for evaluating the efficacy of emerging therapies aimed at improving mental health and overall quality of life in epilepsy patients.

## Methods and analysis

### Study design

**Fig 1:**
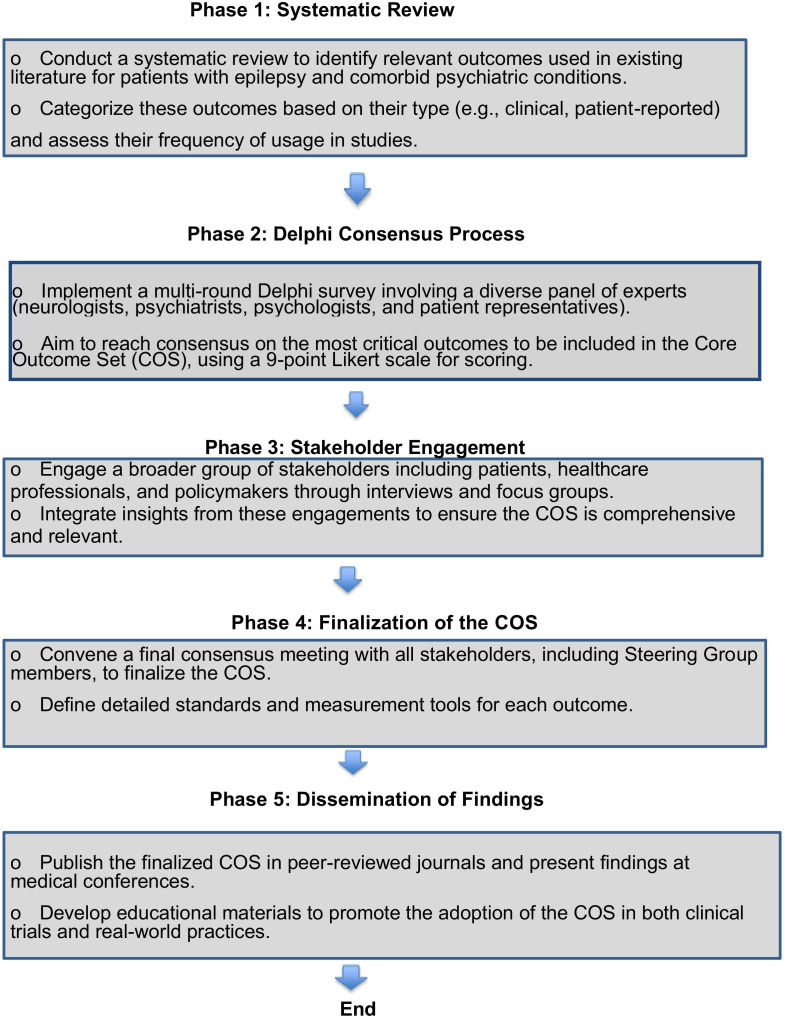
The detailed flow chart of this study.

This protocol outlines a three-phase approach to develop a core outcome set (COS) for post-treatment recovery in adults with epilepsy and comorbid depression or anxiety, following the Core Outcome Set-STAndards for Development (COS-STAD) and Core Outcome Measures in Effectiveness Trials (COMET) [23,24]. It integrates a systematic review, ICF mapping, and a multi-round Delphi process to create a standardized, patient-centered COS addressing neurological and psychiatric recovery. Fig 1 illustrates the workflow. The detailed flow chart of this study is presented in Fig 1.

### Study status and timeline

As of the submission of this protocol, no participant recruitment or data collection has been initiated for the Delphi survey or any other phase of this study. The systematic review (Phase 1) is in the planning stage, with searches scheduled to commence in August 2025. Participant recruitment for the Delphi survey (Phase 2) is expected to begin in October 2025, following the completion of the systematic review and ICF mapping. The first round of the Delphi survey is anticipated to start in December 2025, with subsequent rounds and the consensus meeting planned to conclude by June 2026. The draft survey for the Delphi process has not yet been created, as it will be informed by the systematic review findings, which will identify the initial list of outcome measures. The survey development process, including pilot testing, will adhere to the ethical framework approved by the Institutional Review Board, with any necessary amendments submitted for further review if required. These timelines are provisional and subject to adjustment based on logistical considerations and stakeholder availability.

### Systematic review and ICF mapping

The first phase of our study is a systematic literature review, which is scheduled to begin in August 2025, aimed at identifying outcome measures for adult patients with epilepsy and comorbid mental health conditions. The primary aims of the systematic review are to: (1) identify and categorize outcome domains relevant to post-treatment recovery in adults with epilepsy and comorbid depression or anxiety (e.g., seizure control, mood stability, cognitive function, social participation, quality of life, treatment adherence, and suicide-related outcomes such as suicidal ideation, suicide attempts, and completed suicide), and (2) document specific outcome measures used to assess these domains, including their frequency of use and reported validity. The review will include a variety of study types to capture a broad range of outcomes relevant to post-treatment recovery, such as randomized controlled trials (RCTs) evaluating the efficacy of interventions like antidepressants or cognitive behavioral therapy (CBT) on depression symptoms or suicide risk in epilepsy patients, observational studies assessing quality of life and treatment adherence or suicide-related outcomes in patients with epilepsy and anxiety, and qualitative studies exploring patient-reported outcomes related to social reintegration and mental health management or suicidal behavior. Each study’s relevance is independently assessed by two reviewers, with discrepancies resolved through consensus or involving a third reviewer. Data extraction focuses on study design, participant demographics, outcomes measured, and assessment tools, providing a comprehensive overview that spans physical, mental health, suicide-related and social recovery aspects.

Following the systematic review, the second phase involves linking the identified outcomes to the ICF framework. This process categorizes each outcome under the appropriate ICF domains such as body functions, activities and participation, and environmental factors [25] to ensure that the outcome measures encompass the wide range of impacts that epilepsy and its mental health comorbidities can have on an individual’s life.

### Delphi process

A structured Delphi process will be employed. This Delphi survey will use a secure, user-friendly online platform enabling asynchronous participation and automated response compilation [26]. This technology allows for efficient and real-time updates necessary for maintaining engagement with a diverse, international panel of experts from various disciplines, including neurologists, psychiatrists, psychologists, and patient advocates experienced in the treatment of epilepsy and mental health disorders.

Participants will be asked to rate their level of agreement with statements regarding the inclusion of each outcome measure in the COS, such as “This outcome measure should be included in the core outcome set for post-treatment recovery in adults with epilepsy and comorbid depression or anxiety.” A 9-point Likert scale will be used to assess agreement, with higher scores indicating stronger agreement, as recommended by the Grading of Recommendations Assessment, Development and Evaluation (GRADE) working group and the COMET initiative [24,27]. The scoring will be segmented as follows [28–30] (Table 1):

**Table 1:** The 9-Point Likert Scale for assessing agreement on outcome measure inclusion.

Score Range	Agreement Level	Definition
7–9	Strong Agreement	Measures deemed essential for inclusion in the COS due to their significant impact on treatment and recovery.
4–6	Moderate Agreement	Measures considered beneficial but not essential for inclusion in the COS.
1–3	Disagreement	Measures considered of limited relevance and not necessary for inclusion in the COS.
Unable to score	Uncertain	Option provided for participants who feel they cannot accurately assess the inclusion of a measure due to insufficient information or expertise.

- Scores 7–9: Strong agreement, indicating the outcome measure is deemed essential for inclusion in the COS due to its significant impact on treatment and recovery.
- Scores 4–6: Moderate agreement, suggesting the measure is beneficial but not essential for inclusion in the COS.
- Scores 1–3: Disagreement, indicating the measure is considered less relevant and not necessary for inclusion in the COS.
- “Unable to score”: Provided for participants uncertain about their assessment of an outcome measure due to insufficient information or expertise.

### Sample size

Achieving a representative and valid consensus requires a carefully calculated sample size for the Delphi panel. Based on the COS-STAD recommendations and prior empirical studies [23,29], the study will initially recruit at least 20 participants per stakeholder category, anticipating an attrition rate of approximately 30%. This strategy ensures that, post-attrition, each group retains enough representatives (at least 14 participants) to maintain the integrity and validity of the consensus results. In addition, the participants will represent a broad geographical spread and include diverse professional and experiential backgrounds to encompass a comprehensive array of insights on the treatment and recovery processes in epilepsy with comorbid mental health disorders.

### Data collection and management

Data collection will be managed through the selected online platform, which supports the logistical needs of a multinational Delphi study. This platform will enable the distribution, collection, and systematic analysis of survey data. The setup ensures that data are collected in a manner that is both secure and compliant with international data protection regulations. Responses will be stored on secure servers, and access to the data will be restricted to the research team involved in the study.

All data entries will undergo verification for completeness and consistency. Anonymity will be preserved during data analysis to prevent any bias that might arise from knowing the identity of the respondents. A systematic approach will be adopted for data handling where each stage of data collection and analysis will be documented thoroughly to ensure the process is transparent and reproducible.

Iterative rounds and robust data management practices aim to progressively refine outcome measure selection. Feedback from each round will be analyzed and used to modify subsequent rounds, focusing on areas where consensus is not achieved.

### Survey administration and response collection

The Delphi survey will be administered using Google Forms, chosen for its broad accessibility and ease of use. Prior to launching the main survey, a pilot test will be conducted among a select group of experts to ensure clarity in the presentation of questions, optimal functionality of the online platform, and to gauge the estimated time required for completion. The survey design will emphasize anonymity, formatting questions in a way that responses cannot be traced back to individual participants.

### Consistency and anonymity

To safeguard the anonymity of participants and maintain the integrity of the responses, no personally identifiable information will be collected within the survey. Participants will be assigned a unique identifier known only to the research team. This identifier will facilitate tracking of response rates across the multiple rounds of the Delphi process without linking individual data to specific respondents.

### Data entry and cleaning

Responses from the Google Forms will be automatically consolidated into a Google Sheets document, significantly reducing the potential for manual data entry errors. To ensure data quality, designated members of the research team will carefully review the entries for completeness and consistency. In instances where responses are unclear or ambiguous, participants will be re-contacted for clarification, still ensuring that their anonymity is fully preserved.

### Data synthesis and feedback

Following each round of the Delphi survey, the research team will undertake a thorough analysis of the collected data. This will involve calculating statistical measures including means, medians, interquartile ranges, and percentages of agreement for each proposed outcome measure. The results will be synthesized into a comprehensive summary report that will be devoid of any participant identifiers. This report will be circulated among all participants prior to the subsequent round, highlighting established areas of consensus, points of ongoing disagreement, and integrating new suggestions or comments from the participants.

### Management of iterative rounds

To ensure effective progress and participant engagement throughout the Delphi process, electronic reminders will be sent at predefined intervals to participants who have yet to submit their responses within the allocated timeframe. Adjustments to the survey will be made between rounds based on the evolving consensus: this includes the possible removal of outcome measures that have reached a consensus or the addition of new measures as suggested by the panel.

### Data security

Robust measures will be implemented to ensure the confidentiality and security of all data collected during the Delphi process. Access to the Google Sheets storing participant responses will be strictly limited to authorized research team members only. These precautions are in place to prevent unauthorized access and to ensure that all data handling processes comply with international standards for data protection and privacy.

## Delphi survey, Phase 1

Phase 1 of our study, registered with PROSPERO (registration number: CRD42024576141), involves a systematic review and search strategy to develop a core outcome measure set. We employ an exhaustive search strategy across major databases from their inception to the present. Keywords and Medical Subject Headings (MeSH) terms used include ‘epilepsy,’ ‘depression,’ ‘anxiety,’ ‘post-treatment recovery,’ and ‘outcome measures.’ We will gather a broad spectrum of existing measures, adhering to Preferred Reporting Items for Systematic Reviews and Meta-Analyses (PRISMA) guidelines [31], to facilitate subsequent phases of consensus building through the Delphi method involving international experts.

### Eligibility criteria

Inclusion criteria for our study are defined by patient demographics and study characteristics: studies must involve adult patients (≥18 years) diagnosed with epilepsy and comorbid depression or anxiety, reporting on outcome measures used in post-treatment recovery, including suicide-related outcomes (e.g., suicidal ideation, suicide attempts, and completed suicide). Studies must be published in English within the last 20 years. Exclusion criteria will eliminate studies focused solely on pediatric populations, non-human research, or those not providing original data on relevant outcome measures (Table 2).

**Table 2:** Inclusion and exclusion criteria for the systematic review.

Criteria	Inclusion Criteria	Exclusion Criteria
**Population**	Adults (≥ 18 years) diagnosed with epilepsy and any comorbid depression or anxiety disorders.	Studies involving children or adolescents under 18 years; studies focusing solely on epilepsy without psychiatric comorbidities.
**Study Design**	Randomized controlled trials (RCTs), cohort studies, case-control studies, and cross-sectional studies that report on specific outcomes relevant to epilepsy and mental health recovery.	Editorials, opinion pieces, reviews, and animal studies.
**Outcomes**	Studies must report on both neurological and mental health outcomes, such as seizure frequency, depression scales, anxiety scales, quality of life, and treatment adherence.	Studies reporting only on pharmacokinetics, dose-response, or purely physiological/biochemical parameters without clear clinical outcomes.
**Language**	Studies published in English.	Studies published in languages other than English without an available English translation.
**Publication Date**	Studies published within the last 20 years to ensure contemporary relevance of the data.	Studies published more than 20 years ago.

### Screening and data extraction

Screening will be conducted by two independent reviewers who will assess titles, abstracts, and full texts based on the established eligibility criteria. Disagreements will be resolved through discussion or consultation with a third, senior reviewer. A detailed flowchart will outline the screening process, from the initial number of records identified to the final selection of included studies.

Data extraction will be conducted using a standardized form designed to collect essential information, including study characteristics, population demographics, details of the outcome measures assessed, and the methodologies employed to evaluate these measures. This form will also capture information on predictive models, statistical analyses used, and key findings related to the efficacy and reliability of different outcome measures.

### Statistical analysis and quality assessment

Statistical techniques will be used to synthesize data across studies, focusing on the frequency of use and validity measures of specific outcome measures. Meta-analyses will be conducted where appropriate to synthesize quantitative data on the validity of these outcome measures, provided there is sufficient homogeneity in study designs, populations, and reported outcomes. Specifically, data to be pooled may include psychometric properties, such as sensitivity and specificity of screening tools, reliability coefficients, and responsiveness. Where available, meta-analyses may also pool effect sizes or mean changes in outcome measures (e.g., changes in depression or anxiety scores, seizure frequency or suicide-related outcomes) from intervention studies, such as RCTs evaluating antidepressants or CBT. If heterogeneity in study designs, populations, or reported data precludes meta-analysis, a narrative synthesis will be performed to summarize the most commonly used and validated outcome measures. Critical appraisal of included studies will be performed to assess their quality, using the Cochrane Risk of Bias tool for RCTs and the Newcastle-Ottawa Scale for observational studies, as detailed in the Statistical Analysis and Quality Assessment subsection [32,33].

For missing data, attempts will be made to contact original study authors. If data remain unavailable, appropriate statistical imputation methods will be applied or, alternatively, studies will be excluded from specific analyses based on the extent of missing information.

### Summary and consensus development

The results of the systematic review will inform the subsequent Delphi survey by providing a comprehensive list of outcome measures currently utilized in the field. This list will serve as the basis for the first round of the Delphi process, where selected experts will evaluate the relevance and importance of each outcome measure. Through iterative rounds of surveys, a consensus will be developed on the core outcome measures essential for assessing post-treatment recovery in patients with epilepsy and comorbid mental health disorders.

## Delphi survey, Phase 2

### Phase 2: International online Delphi study

In the second phase of our study, we embark on an international Delphi process to refine and validate the outcome measures identified during the initial systematic review.

### Delphi participants: Stakeholder selection and recruitment

The selection and recruitment of stakeholders will include a diverse spectrum of clinical specialties, geographic representations, and professional experiences to enrich the consensus-building process. We will engage neurologists, psychiatrists, psychologists, epilepsy researchers, mental health nurses, and patient advocates. To ensure a robust and representative international expert panel, participants will be recruited through a multi-faceted approach: (1) identifying authors of relevant peer-reviewed publications on epilepsy and comorbid depression or anxiety from the systematic review (Phase 1); (2) sending invitations through professional associations, such as the International League Against Epilepsy (ILAE) and World Psychiatric Association (WPA), and research networks, such as the COMET Initiative; (3) collaborating with epilepsy and mental health advocacy organizations, such as Epilepsy Action and the International Bureau for Epilepsy (IBE), to recruit patient advocates and caregivers; and (4) using snowball sampling, where initial recruits recommend additional experts, particularly from underrepresented regions, to enhance geographic diversity. Potential participants will receive a detailed invitation letter via email, outlining the study’s purpose, their expected role, and the time commitment. Those expressing interest will be provided with a digital consent form to ensure voluntary participation. Our recruitment strategy ensures a balanced representation from high-, middle-, and low-income countries, acknowledging the influence of varied health care systems and cultural practices on the management of epilepsy and comorbid mental health disorders (Table 3).

**Table 3:** Inclusion criteria for Delphi experts.

Criteria	Description
**Professional Background**	Must be healthcare professionals or researchers with a minimum of 5 years of experience in neurology, psychiatry, psychology, or related fields focusing on epilepsy and/or mental health.
**Experience with Epilepsy and Mental Health**	Experts should have direct clinical experience or research focus on the treatment or study of epilepsy and comorbid mental health disorders such as depression or anxiety.
**Academic Contributions**	Participants should have contributed to the scientific literature on topics relevant to epilepsy and mental health, evidenced by publications in peer-reviewed journals.
**Diversity in Expertise**	The panel should include a diverse range of specialties, including neurologists, psychiatrists, clinical psychologists, and patient advocates to ensure a comprehensive perspective on the outcomes.
**International Representation**	Experts should represent various geographical regions to incorporate a broad spectrum of international practices and insights into the consensus process.
**Language Proficiency**	Experts must be proficient in English to ensure effective communication and understanding during the survey process.

Participants will be selected based on a criterion of having a minimum of 5 years of experience in relevant settings, direct involvement in the care of patients with epilepsy and mental health comorbidities, and a track record of publications or recognized expertise in the field. We will strive for gender and age diversity among the panelists to mitigate potential biases and ensure a broad range of insights. The goal is to recruit at least 60 stakeholders, with a minimum of 20% from low- and middle-income countries, to ensure global applicability of the COS.

### Delphi round 1

The first round of the Delphi survey will focus on evaluating participants’ agreement with the inclusion of each potential outcome measure identified from the systematic review in the context of post-treatment recovery for epilepsy patients with co-occurring depression or anxiety disorders. Participants will be invited via email to access a Google Forms survey, where each measure will be presented alongside a brief description and, where available, supporting evidence from the literature. The inclusion of evidence will be tailored to highlight studies demonstrating the interaction between neurological outcomes and mental health factors. Participants will rate their agreement with statements such as “This outcome measure should be included in the core outcome set” using the 9-point Likert scale. Participants will also have the opportunity to provide qualitative feedback, suggest additional outcome measures specifically relevant to the comorbidities, or elaborate on their ratings. This feedback will be crucial in understanding the multidimensional impacts of epilepsy and its mental health comorbidities on patient recovery. This round will remain open for three weeks, during which participants will receive two reminder emails to maximize response rates. Upon closing, responses will be analyzed, with measures receiving ≥70% scores of 7–9 and ≤15% scores of 1–3 considered to have reached consensus for inclusion, while others may be re-evaluated or excluded based on the collective feedback.

### Delphi round 2

In the second round, participants will receive a summarized report of the results from the first round, highlighting areas of consensus and contention specific to epilepsy and mental health recovery measures. Measures that have reached consensus for inclusion will be confirmed, while those still in dispute will be re-presented for further evaluation with statements such as “This outcome measure should be included in the core outcome set.” New suggestions from the initial round’s open-ended responses, which may include unique insights into the interplay between epilepsy and mental health, will also be included for consideration with similar agreement-based statements.

This round will continue to utilize the 9-point Likert scale, emphasizing critical re-evaluation in light of the group’s feedback. Participants will be encouraged to reconsider their previous scores in the context of the collective feedback and adjust them if necessary, keeping in mind the specific recovery trajectories associated with epilepsy and mental health conditions. Like the first, this round will be open for three weeks, supplemented by reminder emails to ensure timely participation.

### Delphi round 3

The final round is designed to solidify the core set of outcome measures by achieving definitive consensus on measures that uniquely address both neurological and mental health aspects of recovery in epilepsy. Only measures that have not yet reached consensus will be presented, with participants asked to rate their agreement with inclusion statements (e.g., “This outcome measure should be included in the core outcome set”). Participants will reflect on the evolving consensus and address any remaining disagreements.

Should consensus not be reached after three rounds, decisions regarding the inclusion of outcome measures will be based on predefined criteria, including the stability of opinions across rounds, level of ongoing disagreement, and the clinical relevance of each measure to epilepsy patients with depression or anxiety.

## Delphi survey, Phase 3

### Development of the final core outcome measure set

#### Formal consensus meeting.

Upon completion of the Delphi rounds, a pivotal consensus meeting will convene, aimed at finalizing the core outcome measures essential for evaluating post-treatment recovery in epilepsy patients with concurrent depression or anxiety disorders. This critical gathering, occurring either virtually or in person based on participant availability, will incorporate both key contributors from previous rounds and additional experts to cover a wide range of insights. The main goal of this meeting is to thoroughly examine, discuss in depth, and ultimately approve the outcome measures that have come out of the Delphi discussions. We aim to ensure these measures align closely with both the clinical progress and individual recovery paths of the patient group in focus.

During the meeting, facilitators will provide a structured summary of the Delphi results, detailing the levels of consensus achieved, areas of ongoing debate, and the rationale underpinning participants’ decisions. Open discussion sessions will allow attendees to voice differing opinions, propose modifications, and explore alternative perspectives to ensure all viewpoints are considered. Where substantial disagreement persists, voting mechanisms may be utilized to achieve a definitive consensus on each outcome measure.

#### Expert panel endorsement.

Following the consensus meeting, the refined core outcome measures will undergo rigorous scrutiny and endorsement by an expert panel, which includes experts in epilepsy research, neurology, psychiatry, and patient advocacy. This panel will critically assess each measure’s clinical pertinence, feasibility, and potential impact on both patient care and broader healthcare policies, ensuring the set’s alignment with up-to-date clinical practices and its sensitivity to the unique aspects of epilepsy accompanied by psychological disorders.

Furthermore, the expert panel will evaluate the practicalities of implementing these measures in real-world settings. Discussions will span various operational considerations, including standardizing outcome assessments, requisite training for healthcare providers, and identifying potential hurdles to widespread adoption.

The outcome of these phases will be disseminated across academic and professional communities. By promoting standardized application of these measures, this initiative seeks to improve clinical outcomes and research precision for epilepsy patients experiencing comorbid mental health challenges, thereby fostering enhanced care approaches and consistency in clinical trials and treatments.

### Statistical analysis

Upon gathering data from the Delphi rounds, statistical analysis will be conducted on the context of epilepsy with comorbid depression or anxiety using SPSS software (Version 17.0, IBM SPSS Inc.). Descriptive statistics, such as medians, means, standard deviations, and interquartile ranges, will be used to summarize ratings of each outcome measure and to detect patterns pertinent to this patient group across different Delphi rounds. For deeper analysis, non-parametric tests like the Mann–Whitney U-test and Kruskal–Wallis test will be applied, exploring potential variations in responses among different stakeholder groups, such as neurologists, psychiatrists, and patient advocates, and examining differences across the Delphi rounds.

Further analysis will include logistic regression to investigate the factors that aid in achieving consensus on each outcome measure, considering variables like stakeholder type, geographic region, and professional experience. This focused examination will highlight key influences that might affect consensus building, illustrating the complex interplay between clinical viewpoints and contextual variations relevant to the dual challenges of managing epilepsy and mental health disorders.

### Patient and public involvement (PPI)

Recognizing the significance of patient-centered outcomes in epilepsy and mental health management, a dedicated PPI advisory group will be integral to this study. Comprising individuals with firsthand experience of epilepsy and its mental health comorbidities, along with caregivers, this group will provide insights that ensure the core outcomes resonate with patient needs and expectations.

### Ethics and dissemination

The clinical study protocol was presented to the Institutional Review Board (IRB) of the First Affiliated Hospital of Zhengzhou University for approval, which employs an annual review system for ongoing ethical oversight of clinical studies. Written ethical approval was granted in May 2024 (approval number: EC-024-371) based on a detailed protocol outlining the study’s objectives, methodology (systematic review, ICF mapping, and Delphi process), participant recruitment strategy, informed consent process, and data management plan. This preliminary approval covers the overall study design, including the planned Delphi survey, although the draft survey instrument has not yet been developed, as it will be informed by the systematic review findings. The IRB approval allows for the development of the survey instrument, with the condition that any substantial changes, such as the final survey content, will be submitted as an amendment for further review if required by the IRB. Participation in the Delphi survey will be voluntary, and written informed consent will be obtained from all participants prior to their involvement. Participants will receive a digital consent form via email, detailing the study’s purpose, procedures, risks, benefits, and their rights, including the right to withdraw at any time. They will be asked to sign and return the form electronically before accessing the survey. To ensure ethical conduct, a pilot test of the Delphi survey will be conducted to assess clarity and functionality, and any findings from this pilot test that impact participant experience or data collection will be reviewed by the IRB if required. Stringent confidentiality measures, including anonymization of responses and secure data storage, will be implemented to protect participant data.

The dissemination plan is designed to ensure extensive outreach and application of the core outcome measures, targeting both clinical and research communities involved in epilepsy and mental health. The findings will be shared widely, through specialized publications and presentations, and made accessible via a dedicated website and social media, ensuring that the outcomes are easily available and applicable to a broad spectrum of healthcare providers and patients.

### Long-term monitoring and evaluation

A structured plan will monitor the long-term effectiveness of the core outcome measures in real-world settings, focusing on their impact on patient care and recovery in epilepsy with comorbid mental health conditions. This includes follow-up studies, engagement with clinical registries, and periodic surveys to gather feedback from users of the outcome measures, with the goal of continuously refining the outcomes to remain responsive to patient needs and evolving clinical evidence.

## Discussion

This study protocol proposes the development of a core outcome measure set for evaluating post-treatment recovery in patients with epilepsy and comorbid depression or anxiety disorders. This comprehensive approach, underpinned by a rigorous Delphi methodology, holds substantial potential to standardize and enhance the assessment of recovery outcomes, thereby addressing a critical gap in the care of patients with both epilepsy and mental health comorbidities. By establishing a consensus-driven and patient-centered outcome set, the protocol aims to provide clinicians, researchers, and stakeholders with reliable tools for measuring and monitoring recovery outcomes that are both clinically relevant and meaningful to patients, including critical outcomes such as suicide prevention.

A major strength of this protocol lies in its structured approach to identifying and prioritizing recovery outcomes across diverse health dimensions. Drawing from frameworks such as the ICF and leveraging insights from expert stakeholders, the study emphasizes a biopsychosocial perspective, reflecting the complex interplay of neurological, mental health, suicide risk, and social factors affecting recovery in epilepsy patients with mental health comorbidities. This multidimensional framework not only captures traditional clinical endpoints, such as seizure frequency, but also prioritizes quality-of-life measures, emotional well-being, suicide prevention, and functional recovery—outcomes often overlooked in epilepsy studies but highly relevant to patient-centered care.

### Clinical implications and benefits

From a clinical perspective, the development of a core outcome measure set promises significant improvements in personalized care and long-term management for epilepsy patients with comorbid depression or anxiety [34–36]. Through the application of a standardized, multidimensional set of recovery measures, including those related to suicide risk, healthcare providers can more effectively customize treatment plans and follow-up strategies to address the unique needs and recovery paths of each patient [37]. This personalized approach aligns with the growing trend toward holistic and integrated care models, where the evaluation of mental health, suicide prevention, and quality of life is as critical as controlling seizure activity. Improved clarity on which outcomes matter most to patients will also enable clinicians to more effectively engage in shared decision-making, fostering stronger partnerships between patients and providers and ultimately enhancing patient satisfaction and adherence to treatment [38,39].

The core outcome measure set could further inform treatment decisions, enabling clinicians to assess the impact of various interventions not only on seizure control but also on mental health, daily functioning, suicide risk, and social participation. For instance, if a treatment effectively reduces seizure frequency but negatively impacts mood or cognitive function, or increases suicide risk, this outcome measure set would allow clinicians to recognize these trade-offs and consider alternative treatment strategies. This comprehensive view of patient outcomes is particularly valuable in epilepsy care, where treatment decisions often involve balancing seizure control with quality of life considerations and suicide prevention.

In addition to benefiting individual patients, the adoption of a standardized recovery outcome set offers broader healthcare system advantages [40]. Consistent outcome measures, including those addressing suicide, can facilitate comparisons across studies and clinical trials, supporting the generation of high-quality evidence on the efficacy of new treatments or interventions [41,42]. Over time, such a standardized approach could lead to improved healthcare resource allocation by focusing on treatments and strategies that yield the most meaningful outcomes for patients, thus optimizing both clinical effectiveness and cost-efficiency.

### Comparisons with existing studies

This study builds upon existing literature by addressing critical gaps in the standardized evaluation of post-treatment recovery in epilepsy, particularly for patients with comorbid depression or anxiety. While prior research has extensively explored seizure control and recurrence rates, few studies have developed comprehensive outcome sets that reflect the multifaceted recovery needs of this dual-diagnosis population, particularly with respect to suicide risk. For instance, single-focus studies examining the impact of anti-seizure medications on depressive symptoms provide valuable insights but are limited to specific interventions and outcomes, lacking a holistic perspective [43]. Similarly, quality of life assessments, such as the QOLIE scales, contribute to understanding psychosocial impacts but lack specificity for mental health comorbidities, and suicide-related outcomes, making them insufficient for capturing the interplay of neurological and mental health recovery [44].

In contrast to existing COSs, this protocol offers a targeted approach for adults with epilepsy and comorbid depression or anxiety. The EPSET COS for adult epilepsy trials focuses on general outcomes like seizure frequency, adverse events, and overall health, without prioritizing the mental health dimensions or suicide risk critical for patients with depression or anxiety [14]. Likewise, the COS for quality of life in drug-resistant epilepsy emphasizes QOL domains but does not integrate neurological and mental health outcomes specific to mental health comorbidities, such as mood stability or treatment adherence, or suicide prevention [15]. This protocol addresses these limitations by developing a consensus-driven COS that encompasses seizure control, mental health well-being, cognitive function, and social reintegration, and suicide-related outcomes, tailored to the unique needs of this population.

The Delphi-based methodology further distinguishes this study by enabling iterative consensus-building among a diverse, multidisciplinary panel, including neurologists, psychiatrists, psychologists, patient advocates, and caregivers. This approach ensures the COS reflects clinical expertise, patient priorities, including those related to suicide prevention, and global contextual factors, enhancing its applicability across diverse healthcare settings. Unlike single-round or expert-driven methods, the Delphi technique’s iterative refinement fosters a more comprehensive and patient-centered outcome set, aligning with COS-STAD and COMET guidelines. By integrating the ICF framework, this study provides a biopsychosocial lens that complements and extends existing COS efforts, offering a novel tool for research and clinical practice.

### Limitations and considerations

While this study protocol has numerous strengths, it also faces several limitations that merit consideration. First, despite efforts to recruit a geographically and professionally diverse panel, the study may be subject to selection bias. Experts from high-resource settings may be overrepresented in the Delphi rounds, potentially influencing the outcome measures that are prioritized. This could limit the generalizability of the findings to resource-limited settings or regions where healthcare priorities and practices differ. To address this, the study team will endeavor to recruit experts from a range of regions, including low- and middle-income countries, though achieving balanced representation remains challenging.

Another limitation inherent to the Delphi method is the potential for subjective bias. Expert opinion, while valuable, may vary considerably based on individual experience, theoretical orientation, and professional focus. To mitigate this, the Delphi rounds will employ clear inclusion and exclusion criteria, and statistical methods will be used to analyze consensus patterns. However, the consensus-based nature of the methodology does not eliminate the possibility of inherent subjectivity, particularly when balancing diverse perspectives on what constitutes a meaningful recovery outcome.

The iterative nature of Delphi rounds may also contribute to participant fatigue, which could impact engagement and the quality of responses. To counteract this, the study will implement measures to support participants throughout the process, including regular updates on study progress and clear instructions for each round. Additionally, logistical challenges such as coordinating input from participants across different time zones and languages could create delays or affect the timely completion of the Delphi rounds. Careful planning and flexible scheduling will be essential to maintain consistent engagement and minimize dropout rates.

Lastly, the applicability of the core outcome set may be limited by the specific clinical contexts from which the data is derived. Epilepsy treatment practices, available resources, and patient populations can vary widely across healthcare systems, potentially impacting the relevance of the outcome measures to all clinical settings. While the study aims to establish a broadly applicable outcome set, future research will be required to validate its generalizability and predictive validity in diverse settings, particularly in under-resourced environments where care constraints may differ.

## Future directions

The successful development of this core outcome measure set opens avenues for future research and practical applications. Validation studies will be essential to assess the outcome set’s reliability and clinical utility in different healthcare environments and patient populations. Furthermore, implementation studies could investigate the integration of the outcome measure set into clinical workflows, such as electronic health record systems, to facilitate its practical use in monitoring patient progress and guiding treatment adjustments.

Developing user-friendly interfaces and training resources for healthcare providers will also be a key component of the implementation strategy. Ensuring that clinicians understand and are comfortable using the outcome set will be crucial to its adoption and effectiveness in improving patient care. To ensure ongoing relevance, the COS will be reviewed every 5 years to incorporate new evidence and adapt to evolving clinical practices. Longitudinal studies following the implementation of the outcome measure set could provide valuable data on its impact on patient outcomes, quality of life, and healthcare resource use, further contributing to evidence-based epilepsy management practices.

In the broader context, establishing a standardized outcome measure set has the potential to guide international research collaborations, enabling more robust and comparable data collection across studies and clinical trials. Such standardization could support the development of global guidelines for managing epilepsy with mental health comorbidities, fostering consistency and quality in patient care worldwide. Engaging with patient advocacy groups and policymakers will be essential to raise awareness about the importance of standardized outcome measures and encourage their integration into clinical guidelines and quality improvement initiatives.

## Conclusion

This study protocol, utilizing the Delphi method, establishes a crucial framework for standardizing recovery outcomes in patients with epilepsy and associated mental health conditions. It integrates a comprehensive biopsychosocial approach to recovery assessment, emphasizing patient-centered care. The developed core outcome set promises to enhance the evaluation of post-treatment recovery, necessitating future studies for its validation and implementation to significantly improve patient care and outcomes globally.

## Supporting information

S1 File PRISMA-P checklist and COS-STAP checklist. (S1 File. PRISMA-P checklist and COS-STAP checklist.DOC)

S2 File Study protocol approved by the ethics committee. (ZIP)

S3 File Search strategery for Pubmed. (DOCX)
