# Correction: Development of a consensus-based core outcome set for post-treatment recovery in adults with epilepsy and comorbid depression or anxiety: A Delphi and ICF-guided protocol

**DOI:** 10.1371/journal.pone.0336396

**Published:** 2025-11-06

**Authors:** Yan Wang, Zaibang Feng, Zhiyue Guo, Hongxia Xing, Zhaohong Guo, Xinli Zhao, Zubing Mei

There are errors in the author affiliations. The correct affiliations are as follows:

Yan Wang^1^, Zaibang Feng^2^, Zhiyue Guo^3^, Hongxia Xing^1^, Zhaohong Guo^4^, Xinli Zhao^5,6^, Zubing Mei^7,8^

**1** Department of Neurology, The Third Affiliated Hospital of Xinxiang Medical University, Xinxiang, China, **2** Department of Rehabilitation Medicine and Physiotherapy, Changhai Hospital, Naval Military Medical University, Shanghai, China, **3** Department of Physiology and Pathophysiology, School of Basic Medical Sciences, Xinxiang Medical University, Xinxiang, China, **4** School of Health Management, Xinxiang Medical College, Xinxiang, China, **5** Department of Neurosurgery, Zhanjiang Central People’s Hospital, Zhanjiang, Guangdong Province, China, **6** Department of Neurosurgery, The First Affiliated Hospital of Xinxiang Medical University, Zhengzhou, Henan Province, China, **7** Department of Anorectal Surgery, Shuguang Hospital, Shanghai University of Traditional Chinese Medicine, Shanghai, China, **8** Anorectal Disease Institute of Shuguang Hospital, Shanghai, China.
