# Supplementary material for: Development of a consensus-based core outcome set for post-treatment recovery in adults with epilepsy and comorbid depression or anxiety: A Delphi and ICF-guided protocol
Source: PLoS One. 2025 Aug 22;20(8):e0330617. doi: 10.1371/journal.pone.0330617 (PMC12373203; doi:10.1371/journal.pone.0330617)
Supplement: S1 File — (S1 File. PRISMA-P checklist and COS-STAP checklist.DOC) [file pone.0330617.s001.doc]

**PRISMA-P (Preferred Reporting Items for Systematic review and Meta-Analysis Protocols) 2015 checklist: recommended items to address in a systematic review protocol***

| Section and topic | Item No | Checklist item | Reported on page number |
| --- | --- | --- | --- |
| ADMINISTRATIVE INFORMATION | | |  |
| Title: |  |  |  |
| Identification | 1a | Identify the report as a protocol of a systematic review | 5 |
| Update | 1b | If the protocol is for an update of a previous systematic review, identify as such | N/A |
| Registration | 2 | If registered, provide the name of the registry (such as PROSPERO) and registration number | 7 |
| Authors: |  |  |  |
| Contact | 3a | Provide name, institutional affiliation, e-mail address of all protocol authors; provide physical mailing address of corresponding author | 1-2 |
| Contributions | 3b | Describe contributions of protocol authors and identify the guarantor of the review | 3 |
| Amendments | 4 | If the protocol represents an amendment of a previously completed or published protocol, identify as such and list changes; otherwise, state plan for documenting important protocol amendments | N/A |
| Support: |  |  |  |
| Sources | 5a | Indicate sources of financial or other support for the review | 2 |
| Sponsor | 5b | Provide name for the review funder and/or sponsor | N/A |
| Role of sponsor or funder | 5c | Describe roles of funder(s), sponsor(s), and/or institution(s), if any, in developing the protocol | N/A |
| INTRODUCTION | | |  |
| Rationale | 6 | Describe the rationale for the review in the context of what is already known | 5-8 |
| Objectives | 7 | Provide an explicit statement of the question(s) the review will address with reference to participants, interventions, comparators, and outcomes (PICO) | 6 |
| METHODS | | |  |
| Eligibility criteria | 8 | Specify the study characteristics (such as PICO, study design, setting, time frame) and report characteristics (such as years considered, language, publication status) to be used as criteria for eligibility for the review | 12 |
| Information sources | 9 | Describe all intended information sources (such as electronic databases, contact with study authors, trial registers or other grey literature sources) with planned dates of coverage | 12 |
| Search strategy | 10 | Present draft of search strategy to be used for at least one electronic database, including planned limits, such that it could be repeated | Supplementary Material |
| Study records: |  |  |  |
| Data management | 11a | Describe the mechanism(s) that will be used to manage records and data throughout the review | 15-16 |
| Selection process | 11b | State the process that will be used for selecting studies (such as two independent reviewers) through each phase of the review (that is, screening, eligibility and inclusion in meta-analysis) | 18 |
| Data collection process | 11c | Describe planned method of extracting data from reports (such as piloting forms, done independently, in duplicate), any processes for obtaining and confirming data from investigators | 19 |
| Data items | 12 | List and define all variables for which data will be sought (such as PICO items, funding sources), any pre-planned data assumptions and simplifications | NA |
| Outcomes and prioritization | 13 | List and define all outcomes for which data will be sought, including prioritization of main and additional outcomes, with rationale | 6-11 |
| Risk of bias in individual studies | 14 | Describe anticipated methods for assessing risk of bias of individual studies, including whether this will be done at the outcome or study level, or both; state how this information will be used in data synthesis | 20 |
| Data synthesis | 15a | Describe criteria under which study data will be quantitatively synthesised | 20 |
|  | 15b | If data are appropriate for quantitative synthesis, describe planned summary measures, methods of handling data and methods of combining data from studies, including any planned exploration of consistency (such as I^2^, Kendall’s τ) | N/A |
|  | 15c | Describe any proposed additional analyses (such as sensitivity or subgroup analyses, meta-regression) | N/A |
|  | 15d | If quantitative synthesis is not appropriate, describe the type of summary planned | N/A |
| Meta-bias(es) | 16 | Specify any planned assessment of meta-bias(es) (such as publication bias across studies, selective reporting within studies) | N/A |
| Confidence in cumulative evidence | 17 | Describe how the strength of the body of evidence will be assessed (such as GRADE) | 13 |

*** It is strongly recommended that this checklist be read in conjunction with the PRISMA-P Explanation and Elaboration (cite when available) for important clarification on the items. Amendments to a review protocol should be tracked and dated. The copyright for PRISMA-P (including checklist) is held by the PRISMA-P Group and is distributed under a Creative Commons Attribution Licence 4.0.**

*From: Shamseer L, Moher D, Clarke M, Ghersi D, Liberati A, Petticrew M, Shekelle P, Stewart L, PRISMA-P Group. Preferred reporting items for systematic review and meta-analysis protocols (PRISMA-P) 2015: elaboration and explanation. BMJ. 2015 Jan 2;349(jan02 1):g7647.*

**The Core Outcome Set-STAndardised Protocol Items (COS-STAP) checklist (protocol).**

| **COS-STAP Item** | **Description** | **Manuscript Compliance** | **Details and Suggestions** |
| --- | --- | --- | --- |
| **1. Title** | Identify the report as a protocol for a COS. | **Fully Reported** | The title, "Development of a Consensus-Based Core Outcome Set for Post-Treatment Recovery in Adults with Epilepsy and Comorbid Depression or Anxiety: A Delphi and ICF-Guided Protocol," clearly identifies it as a COS protocol, specifying the population and methods (Delphi, ICF). |
| **2. Abstract** | Structured summary including objectives, methods, and anticipated outcomes. | **Fully Reported** | The abstract includes Introduction (objectives, gap), Methods and Analysis (systematic review, ICF mapping, Delphi details), Discussion (gap addressed, limitations), and Conclusion (anticipated outcomes). It is concise (321 words) and covers all required elements. |
| **3. Background** | 3a. Health condition(s) and population(s) covered by the COS. | **Fully Reported** | The Introduction specifies epilepsy with comorbid depression or anxiety in adults (≥18 years), citing prevalence (30–50%) and impacts (e.g., quality of life, adherence) [References 1, 4–7]. |
|  | 3b. Explanation of why a COS is needed and intended uses. | **Fully Reported** | The Introduction highlights the lack of a COS for this dual-diagnosis population, limitations of existing tools (e.g., QOLIE), and intended uses (cross-study comparability, personalized care) [References 14–16]. The research question is explicit. |
|  | 3c. Existing outcome measurement knowledge in the health area. | **Fully Reported** | The Introduction critiques existing COSs (EPSET, drug-resistant epilepsy QOL) and tools (QOLIE), noting their lack of psychiatric focus [References 14–16]. It establishes the gap in standardized outcomes for depression/anxiety. |
| **4. Objectives** | Primary objective(s) of the COS development. | **Fully Reported** | The Introduction states: “This study addresses the research question: What are the essential outcomes for evaluating post-treatment recovery in adults with epilepsy and comorbid depression or anxiety, as agreed upon by international stakeholders?” The objective is clear. |
| **5. Scope** | 5a. Health condition(s) and population(s). | **Fully Reported** | The Introduction and Methods specify adults with epilepsy and comorbid depression/anxiety [Table 2]. |
|  | 5b. Interventions targeted by the COS. | **Fully Reported** | The manuscript broadly refers to “post-treatment recovery” and explicitly list all interventions for epilepsy and depression/anxiety (e.g., antiepileptic drugs, antidepressants, behavioral therapies). |
|  | 5c. Settings for intended use of the COS. | **Fully Reported** | The Introduction and Discussion mention clinical trials and routine practice globally, with emphasis on diverse settings [References 21–22, Discussion]. No changes needed. |
| **6. Methods** | 6a. Study design and stages for COS development. | **Fully Reported** | The Methods detail a three-phase approach: systematic review, ICF mapping, and Delphi process, with a flowchart (Figure 1). COS-STAD and COMET are cited [References 23–24]. |
|  | 6b. Identification of outcomes (e.g., literature reviews). | **Fully Reported** | The systematic review (Phase 1) is described, including databases (PubMed, Embase, Cochrane Library), PRISMA adherence, and eligibility criteria (Table 2) [Reference 31]. |
|  | 6c. Consensus process (e.g., Delphi, nominal group). | **Fully Reported** | The Delphi process (Phase 3) is detailed: three rounds, 9-point Likert scale, consensus criteria (≥70% for 7–9, ≤15% for 1–3), and a consensus meeting [References 24, 27–30]. Google Forms and anonymity measures are specified. |
|  | 6d. Criteria for including/retaining outcomes in the COS. | **Fully Reported** | Consensus criteria are defined (≥70% scoring 7–9, ≤15% scoring 1–3), with median/IQR for Round 1 retention and voting for unresolved measures [Methods: Delphi Round 1–3]. |
| **7. Stakeholders** | 7a. Groups to be involved (e.g., clinicians, patients). | **Fully Reported** | Stakeholders include neurologists, psychiatrists, psychologists, patients, caregivers, and advocates, with ≥60 participants and ≥20% from low/middle-income countries (Table 3) [Methods: Delphi Participants]. |
|  | 7b. Methods for recruiting stakeholders. | **Fully Reported** | Recruitment via professional networks and patient organizations, with inclusion criteria (≥5 years’ experience, publications) [Table 3]. |
| **8. Patient/Public Involvement** | How patients/public are involved in COS development. | **Fully Reported** | A 10-member PPI advisory group (patients, caregivers) is described, ensuring patient-centered outcomes [Methods: Patient and Public Involvement]. |
| **9. Ethics** | Ethical approval and consent procedures. | **Fully Reported** | Ethics approval (EC-024-371, May 2024) and consent processes are detailed, with confidentiality measures [Methods: Ethics and Dissemination, S2 File]. |
| **10. Data Management** | How data will be collected, stored, and analyzed. | **Fully Reported** | Data collection via Google Forms, storage on secure servers, and analysis using SPSS v17.0 (Mann-Whitney U, Kruskal-Wallis, logistic regression) are described [Methods: Data Collection and Management, Statistical Analysis]. No changes needed. |
| **11. Dissemination** | Plans for disseminating the COS. | **Fully Reported** | Dissemination via publications, presentations, a website, and social media is planned, targeting clinical/research communities [Methods: Ethics and Dissemination]. No changes needed. |
| **12. Project Management** | 12a. Investigators and roles. | **Fully Reported** | Authors’ roles are listed (CRediT statement), with co-first (Wang, Feng) and co-corresponding (Zhao XL, Mei ZB) authors specified. |
|  | 12b. Funding sources. | **Fully Reported** | No funding is declared [Funding]. |
|  | 12c. Conflicts of interest. | **Fully Reported** | No conflicts are declared [Declaration of Competing Interest]. |
| **13. Updates** | Plans for updating the COS post-development. | **Fully Reported** | The Discussion mentions validation and implementation studies, also clearly stating periodic COS review (e.g., every 5 years) based on new evidence. |

*From: Kirkham, J.J., Gorst, S., Altman, D.G. et al. COS-STAR: a reporting guideline for studies developing core outcome sets (protocol). Trials 16, 373 (2015). https://doi.org/10.1186/s13063-015-0913-9*
