# Supplementary material for: Development of a consensus-based core outcome set for post-treatment recovery in adults with epilepsy and comorbid depression or anxiety: A Delphi and ICF-guided protocol
Source: PLoS One. 2025 Aug 22;20(8):e0330617. doi: 10.1371/journal.pone.0330617 (PMC12373203; doi:10.1371/journal.pone.0330617)
Supplement: S2 File — (ZIP) [file pone.0330617.s002.zip › S2 File. Study protocol in Chinese.pdf]

伴有抑郁或焦虑的癫痫患者治疗后康复的核心结局指标集的开发和国际共识:一项定性研究

主要研究者： 赵新利，新乡医学院第一附属医院 神经外科

电子邮件： 13782510911@163.com

地址： 河南新乡卫辉健康路88号

版本： 1.0

日期： 2024年5月27日

摘要

引文：

癫痫患者常同时患有抑郁和焦虑障碍，这种共病不仅加重了治疗的复杂性，也显著影响了康复的结局评估。对于这一人群的治疗后康复评估，需要一套标准化且全面的结局指标集，既能涵盖神经系统层面的恢复，也能反映精神心理维度的改善。然而，目前现有的评估方法往往忽视了这种双重疾病状态的特点，从而难以准确评估长期的康复情况。国际功能、残疾和健康分类（ICF）框架提供了一种多维度的评估方式，可以支持专门针对这种双重诊断人群的核心结局集的开发。本研究旨在开发一套针对癫痫合并抑郁或焦虑障碍患者治疗后康复的核心结局指标集，以实现跨研究的结局对比，同时通过结合癫痫学和心理学的康复维度，显著优化治疗策略。

方法与分析：

本研究将采用三阶段流程，基于共识开发核心结局指标集：

1. **系统综述：** 在主要文献数据库（如PubMed、Embase和Cochrane Library）中，系统检索现有文献中针对癫痫及其共病精神障碍患者的相关结局指标，以确定现有研究中使用的结局指标。
2. **ICF框架映射：** 将检索到的结局指标映射到ICF框架，以确保结局指标的全面性和多维度覆盖。该框架可帮助涵盖神经学和精神心理学的双重领域。
3. **Delphi调查法：** 采用多轮Delphi专家问卷调查，基于9分Likert评分量表，邀请来自多学科领域（包括神经学家、精神科医生及患者代表）的国际专家组参与讨论和评分，逐步达成共识。通过这一迭代过程，最终

确定核心指标集，涵盖如认知功能、情绪稳定性和生活质量等关键康复领域，并确保指标集在不同医疗背景下的适用性。

讨论：

通过开发一套标准化的核心结局指标集，本研究填补了当前癫痫患者及其共病抑郁或焦虑障碍治疗后康复评估领域的空白。采用ICF框架的多维度评估方式，确保康复过程的评价能够从生物-心理-社会的综合视角出发，涵盖影响患者整体健康状况的神经学和心理学层面。利用Delphi方法建立的核心结局集将为未来的临床研究提供坚实的基础，有助于在国际范围内统一结局报告的标准，从而促进针对性治疗干预措施的发展。

研究方案

1. 研究背景

癫痫是全球最常见的神经系统疾病之一，约有5000万人受到其影响[1]。该病以反复发作的非诱发性癫痫发作为特征，发作源于大脑中异常的电活动。除去与癫痫发作相关的症状负担外，许多癫痫患者还面临各种心理和社会心理挑战，这些问题进一步复杂化了治疗过程并影响预后[2，3]。在这些挑战中，伴随的精神健康障碍，尤其是抑郁症和焦虑症，具有高度的流行性，并显著增加了疾病的总体负担[4]。估计约30%至50%的癫痫患者同时患有重度抑郁症或焦虑症，而这些精神障碍被认为加重了癫痫对患者生活质量、社会功能及治疗依从性的影响[5，6]。这种共病状态通常会导致较差的治疗效果、增加的医疗资源消耗和癫痫治疗效果的下降[7]。

抑郁症和焦虑症对癫痫患者的影响深远且多方面。例如，抑郁症已被发现与癫痫患者自杀意念和自杀尝试的发生率较高相关[8，9]，这凸显了对这些患者进行全面、积极心理健康管理的迫切需求。另一方面，焦虑症可能由于生理性兴奋状态增加而加剧癫痫发作的频率，进一步削弱患者有效管理病情的能力[10]。此外，精神健康障碍还会干扰治疗依从性[11，12]，因为抑郁或焦虑的患者可能表现出较低的依从性，导致他们不愿按照规定服用抗癫痫药物或进行生活方式改变。尽管现有多种癫痫治疗手段（包括药物治疗、神经调节和行为干预等），但缺乏一套标准化、全面的评估工具来衡量患者的康复结局，依然是临床实践中的一个重要障碍。没有这些工具，医疗提供者无法有效地评估这一复杂群体治疗后康复的全貌。

目前癫痫治疗后康复的评估方法通常过于依赖自我报告的临床症状以及医疗提供者的主观评估[13]。尽管这些方法能提供一些患者状况的洞见，

但它们常常缺乏标准化、客观性和可重复性。此外，传统的评估方法往往过于侧重癫痫发作的频率和严重性，忽视了康复过程中更加广泛的心理社会功能和生活质量维度，这些维度对于伴有精神健康障碍的患者尤其重要。例如，涉及生活质量、情绪稳定性、认知功能和社会重返等方面的评估指标，往往在传统工具中缺乏充分体现，尽管这些方面在患者康复的整体评估中具有重要意义。因此，迫切需要一种更为结构化和标准化的结局指标集，以反映癫痫患者在伴随抑郁或焦虑症的情况下康复的复杂性。

核心结局指标集的开发为解决这些挑战提供了可能，它通过建立标准化的基准，可以在临床试验和日常实践中进行广泛应用。国际功能、残疾和健康分类（ICF）框架由世界卫生组织提出，提供了一个全面理解健康和残疾的模型[14, 15]。ICF框架关注生物学、心理学和社会因素之间的相互作用，因此它为开发一个考虑多维度健康状况的核心结局集提供了理想基础[16, 17]。通过将康复结局映射到ICF的不同领域——如身体健康、心理福祉和社会参与，研究人员可以创建一个更为全面的工具，更好地满足癫痫合并精神健康障碍患者的需求。在癫痫研究中采用ICF基础的结局指标集，将增强监测不同患者群体和医疗环境下康复的能力，最终促进治疗策略的优化，提升长期预后[18, 19]。

本研究方案旨在开发一套国际共识的核心结局指标集，用于评估癫痫及合并抑郁或焦虑障碍患者的治疗后康复。为实现这一目标，我们将采用Delphi方法，这是一种结构化的、迭代的过程，能够通过系统化汇总专家意见达成共识。本研究的预期结果是构建一个验证过的、基于共识的核心结局指标集，作为评估癫痫及共病抑郁或焦虑障碍患者治疗后康复的标准化工具。通过关注健康的身体、心理和社会维度，这一工具将帮助临床医生和研究人员全面捕捉患者康复的多个方面，超越单纯的癫痫发作控制。

预计这一标准化结局集将促进跨研究的比较，支持系统评价中的证据整合，并为个性化治疗方案的制定提供指导。此外，它还将成为评估新兴治疗手段（尤其是改善癫痫患者精神健康和整体生活质量的疗法）效果的宝贵资源。

2. 第一部分：Delphi调查的设计与实施

本研究采用Delphi方法来开发癫痫合并抑郁或焦虑症患者康复评估的核心结局指标集。这一方法的选择基于其能够系统地收集和综合专家意见的优势，特别适用于在医学和健康领域形成广泛共识的需求。

初始准备和文献回顾

研究的起始阶段集中在广泛的文献回顾上，目的是收集和评估当前关于癫痫及其心理并发症治疗后康复的相关文献。这包括系统地搜索PubMed、Embase和Cochrane Library等医学数据库，筛选关键词如“癫痫”、“抑郁”、“焦虑”、“治疗后康复”和“结果测量”。通过这一过程，我们能够识别出已被广泛记录的治疗效果和康复评估指标，这些成果为后续Delphi调查的设计提供了科学基础。

文献筛选和评估按照PRISMA（Preferred Reporting Items for Systematic Reviews and Meta-Analyses）指南进行[20]，以确保方法的透明性和可重复性。证据质量将通过使用Cochrane偏倚风险工具评估随机对照试验，以及使用纽卡斯尔-渥太华量表评估观察性研究[21，22]。通过这一阶段的工作，团队建立了初步的结果指标清单，这些指标被认为对评估癫痫及其心理并发症的治疗效果至关重要。

Delphi调查的设计与试点测试

在建立初步指标清单后，我们设计了Delphi调查问卷，首先在一个小型的专家团队中进行试点测试。试点测试主要是为了验证问卷的内容是否清晰，是否能够有效地衡量专家对各治疗效果指标的看法。此外，试点也帮助我们优化问卷的格式和操作流程，确保在更广泛的专家群体中实施时可以顺利进行。

调查的实施

Delphi调查通过谷歌表格在线进行，参与者包括神经学家、精神病学家、心理学家以及经验丰富的患者代表。我们利用匿名方式收集数据，以鼓励参与者提供开放而诚实的反馈。每位专家对结果指标使用9点Likert量表进行评分，其中1-3分表示指标不重要，4-6分表示重要但非关键，7-9分表示非常重要且必须包括在核心结局指标集中。

我们采用加密技术来保护数据传输和存储的安全，确保参与者的意见保持匿名。这种数据保护措施不仅符合伦理标准，也增强了调查的有效性，因为参与者能够在保证个人信息安全的情况下，更自由地表达真实意见。

样本量

达成代表性和有效共识需要为Delphi小组精心计算样本大小。根据核心成果集-开发标准的建议和以前的经验研究，研究最初将至少招募每个利益相关者类别的20名参与者，预计流失率约为30%。这种策略确保每个组在流失后仍有足够的代表（至少14名参与者），以维持共识结果的完整性和有效性。此外，参与者将代表广泛的地理分布并包括不同的专业和经验背景，以涵盖关于癫痫治疗和康复过程的全面见解。

调查的迭代过程

根据Delphi方法的标准，我们预计进行至少三轮调查，以逐步精炼和验证结果指标。每轮调查后，研究团队将对收集到的数据进行详细分析，计算每个指标的平均得分和一致性指数。基于这些数据，指标可能会被修改或合并，以确保它们的相关性和重要性最大化。

通过这种结构化和迭代的过程，我们期望能够最终确定一个科学严谨且实用的核心结局指标集。这些指标不仅将用于评估治疗效果，也将帮助临床医生、研究人员和患者更好地理解和改善癫痫及其心理并发症的治疗

方案。通过Delphi方法的精细操作，本研究将能够为癫痫合并心理障碍的康复评估提供一个坚实而全面的基础。

3. 数据管理与参与者的匿名性保护

在本研究中，采用Delphi调查法对癫痫合并抑郁或焦虑症患者的康复评估指标进行开发和验证，对数据管理和参与者匿名性的保护尤为重要。这不仅有助于保证调查的科学性和客观性，还是对参与者隐私权的基本尊重。

数据匿名化与加密技术的应用

为了最大限度地保护参与者的匿名性，本研究从设计之初就采用了严格的数据匿名化措施。每位参与者在调查开始时被分配一个随机生成的唯一识别码，这一代码与其个人身份信息完全脱钩。所有调查数据的收集、存储和处理均使用这一代码进行标识，确保个人信息的匿名性。

此外，为保证数据传输过程中的安全性，研究团队采用了先进的加密技术。所有在线调查表单的传输均通过安全套接层（SSL）加密，这是一种广泛用于保护网络通信和敏感数据的安全技术。SSL加密不仅确保了数据在传输过程中的安全性，也防止了未经授权的访问和数据泄露。

数据的收集和存储

调查数据的收集通过自动化的Google表格进行，这种方式不仅方便快捷，而且有助于减少因人为操作引发的数据错误。一旦数据被录入，它们就被自动保存在受密码保护的服务器上，只有授权的研究团队成员才能访问这些信息。

研究团队对数据的访问和使用也进行了严格的规定。每位团队成员在获取访问权限前必须签署保密协议，明确他们在数据处理过程中的责任和义务。此外，所有涉及数据分析的操作都必须在保证数据匿名性的前提下进行。

数据完整性的检查和监控

为了确保收集数据的准确性和完整性，研究团队制定了一系列的数据审核流程。在每一轮Delphi调查后，数据团队会进行数据完整性的检查，包括验证数据的完整性、一致性和逻辑性。任何发现的错误或不一致都将立即更正，确保分析的准确性。

这种严格的数据管理机制不仅保护了参与者的匿名性，也增强了调查数据的可靠性和研究结果的有效性。通过这些措施，研究团队可以确信，所得到的数据能够真实反映专家的意见和建议，进而为癫痫及其心理并发症的治疗和康复提供科学、有效的评估指标。

4.利益相关者的广泛参与和成果的发布与实施

在本研究中，我们注重确保广泛的利益相关者参与，以及研究成果的有效发布和实施。这不仅增加了研究结果的可接受度和实用性，还有助于将这些成果转化为实际的临床实践改进。

利益相关者的识别与参与

首先，确定和纳入各方利益相关者是本研究成功的关键。我们的利益相关者包括神经科医生、精神科医生、心理学家、康复专家、患者及其家属。通过识别这些群体的具体需求和期望，研究团队能够更好地设计调查问卷，并确保调查结果反映出广泛的专业和患者视角。

为了有效地纳入这些利益相关者，我们采取了多种策略。一方面，我们与专业协会合作，通过他们的网络联系到专业人士，并邀请他们参与Delphi调查。另一方面，我们也通过患者支持团体和社交媒体平台，接触到更广泛的患者和家属群体，确保他们的声音和需求得到充分考虑。

成果的发布

在Delphi调查完成并形成最终的核心结局指标集后，研究团队将着手准备成果的发布。这包括撰写详细的研究报告和论文，以及在专业会议上进行口头和海报展示。这些成果的发布旨在向医疗界和公众传达研究的方法、发现和推荐，增加研究的可见性和影响力。

我们计划通过开放获取的医学期刊发布研究论文，确保所有利益相关者，无论是医疗专业人士还是患者，都能无障碍地访问这些重要信息。此外，研究团队还将利用数字媒体，如博客和社交媒体，以更为通俗的语言解释研究成果，扩大其教育和倡导的作用。

成果的实施

发布研究成果之后，接下来的关键步骤是实施这些成果，将研究所得的核心结局指标集应用于临床实践。为此，我们将与医疗机构和专业团体合作，开发培训材料和指南，帮助医生和康复专家理解和采用这些新的评估工具。

此外，研究团队计划监测指标集的实施效果，通过定期收集反馈和实施数据，评估其在实际医疗环境中的有效性和可行性。这些信息将用于不断优化和调整指标集，确保它们能够真正改善癫痫及其心理并发症患者的康复过程。

通过这样全面而深入的方法，本研究不仅提供了一套科学验证的康复评估工具，也促进了广泛利益相关者的有效参与和深度合作，从而推动了癫痫及其心理并发症治疗领域的持续进步和创新。

参考文献

1. Vaughan, K.A., et al., An estimation of global volume of surgically treatable epilepsy based on a systematic review and meta-analysis of epilepsy. J Neurosurg, 2019. 130(4): p. 1127-1141.
2. Keikelame, M.J., et al., Psychosocial challenges affecting the quality of life in adults with epilepsy and their carers in Africa: A review of published evidence between 1994 and 2014. Afr J Prim Health Care Fam Med, 2017. 9(1): p. e1-e5.
3. Mula, M. and J.W. Sander, Psychosocial aspects of epilepsy: a wider approach. BJPsych Open, 2016. 2(4): p. 270-274.
4. Keezer, M.R., S.M. Sisodiya, and J.W. Sander, Comorbidities of epilepsy: current concepts and future perspectives. Lancet Neurol, 2016. 15(1): p. 106-15.
5. Lu, E., et al., Systematic Literature Review of Psychiatric Comorbidities in Adults with Epilepsy. J Clin Neurol, 2021. 17(2): p. 176-186.
6. Pham, T., et al., The prevalence of anxiety and associated factors in persons with epilepsy. Epilepsia, 2017. 58(8): p. e107-e110.
7. Patel, R.S., et al., Psychiatric Comorbidities and Outcomes in Epilepsy Patients: An Insight from a Nationwide Inpatient Analysis in the United States. Cureus, 2017. 9(9): p. e1686.
8. Friedman, D., et al., Depressive symptoms and suicidality among individuals with epilepsy enrolled in self-management studies: Results from the US Centers for Disease Control and Prevention Managing Epilepsy Well (MEW) Network. Epilepsy Behav, 2018. 87: p. 235-240.
9. Nigussie, K., et al., Magnitude and associated factors of suicidal ideation and attempt among people with epilepsy attending outpatient treatment at primary public hospitals in northwest Ethiopia: a multicentre cross-sectional study. BMJ Open, 2021. 11(1): p. e043227.
10. Dehn, L.B., et al., Relationships of depression and anxiety symptoms with seizure frequency: Results from a multicenter follow-up study. Seizure, 2017. 53: p. 103-109.
11. Roca, M., et al., Adherence to antidepressant treatment in depressive patients with comorbid psychiatric disorders. European Psychiatry, 2011. 26(S2): p. 1277-1277.
12. Litz, M. and D. Leslie, The impact of mental health comorbidities on adherence to buprenorphine: A claims based analysis. Am J Addict, 2017. 26(8): p. 859-863.
13. Mücke, F.J., et al., Discrepancy between subjective and objective memory change after epilepsy surgery: Relation with seizure outcome and depressive symptoms. Front Neurol, 2022. 13: p. 855664.
14. Huang, Y., et al., Application of the International Classification of Functioning, Disability and Health (ICF) in dementia research and practice: A scoping review. Aging Ment Health, 2023. 27(2): p. 357-371.
15. Leonardi, M., et al., 20 Years of ICF-International Classification of Functioning, Disability and Health: Uses and Applications around the World. Int J Environ Res Public Health, 2022. 19(18).
16. Bornbaum, C.C., et al., A critical exploration of the International Classification of Functioning, Disability, and Health (ICF) framework from the perspective of oncology: recommendations for revision. J Multidiscip Healthc, 2013. 6: p. 75-86.
17. Ewert, T., et al., Validation of the International Classification of Functioning Disability and Health framework using multidimensional item response modeling. Disabil Rehabil, 2010. 32(17): p. 1397-405.
18. Crudgington, H., et al., Core Health Outcomes in Childhood Epilepsy (CHOICE): Development of a core outcome set using systematic review methods and a Delphi survey consensus. Epilepsia, 2019. 60(5): p. 857-871.
19. Noble, A.J. and A.G. Marson, Which outcomes should we measure in adult epilepsy trials? The views of people with epilepsy and informal carers. Epilepsy Behav, 2016. 59: p. 105-10.
20. Page, M.J., et al., PRISMA 2020 explanation and elaboration: updated guidance and exemplars for reporting systematic reviews. Bmj, 2021. 372: p. n160.
21. Higgins, J.P., et al., The Cochrane Collaboration's tool for assessing risk of bias in randomised trials. Bmj, 2011. 343: p. d5928.
22. Stang, A., Critical evaluation of the Newcastle-Ottawa scale for the assessment of the quality of nonrandomized studies in meta-analyses. Eur J Epidemiol, 2010. 25(9): p. 603-5.23.
