# Supplementary material for: Development of a consensus-based core outcome set for post-treatment recovery in adults with epilepsy and comorbid depression or anxiety: A Delphi and ICF-guided protocol
Source: PLoS One. 2025 Aug 22;20(8):e0330617. doi: 10.1371/journal.pone.0330617 (PMC12373203; doi:10.1371/journal.pone.0330617)
Supplement: S2 File — (ZIP) [file pone.0330617.s002.zip › S2 File. Study protocol in English.pdf]

Development and International Consensus on a Core Outcome Measure Set for Post-Treatment Recovery in Patients with Epilepsy and Comorbid Depression or Anxiety Disorders: A Study Protocol

Principle Investigator: Prof. Xinli Zhao, Department of Neurosurgery, The First Affiliated Hospital of Xinxiang Medical University, Xinxiang, Henan Province, China.

E-mail: 13782510911@163.com

Address: No. 88 Jiankang Road, Weihui, Xinxiang, Henan, 453100 China

Version: 1.0

Date: May 27 , 2024

Abstract

Introduction

Epilepsy patients frequently experience coexisting depression and anxiety disorders, which complicate treatment and impact recovery outcomes. Assessing post-treatment recovery in this population requires a standardized, comprehensive set of outcome measures that considers both neurological and psychiatric dimensions. Existing measures often overlook the dual nature of these conditions, making it challenging to evaluate long-term recovery accurately. The International Classification of Functioning, Disability, and Health (ICF) framework provides a multidimensional approach that can support the development of a core outcome set specific to this dual-diagnosis population. This study aims to develop a specialized core outcome measure set, uniquely tailored for evaluating post-treatment recovery in patients with epilepsy and comorbid depression or anxiety. This set is designed to facilitate robust cross-study comparisons and significantly enhance care strategies by incorporating both epileptological and psychological aspects of recovery.

Methods and analysis

This study will follow a three-phase process to develop a consensus-based outcome measure set: (1) Conduct a systematic review to identify relevant outcomes used in existing literature for epilepsy with comorbid psychiatric disorders, utilizing major databases such as PubMed, Embase, and the Cochrane Library, (2) Map identified outcomes to the ICF framework to ensure comprehensive and holistic coverage, and (3) Utilize a multi-round Delphi survey employing a 9-point Likert scale to achieve international consensus among a panel of multidisciplinary experts, including neurologists, psychiatrists, and patient representatives. This iterative process will refine the measure set to capture critical domains of recovery, such as cognitive function, mood stability, and quality of life, ensuring relevance across different healthcare contexts.

Discussion

By developing a standardized core outcome measure set, this study addresses the current gap in post-treatment assessment for epilepsy patients with comorbid depression or anxiety disorders. The ICF-based approach ensures that recovery is evaluated from a biopsychosocial perspective, accommodating both neurological and psychological aspects that affect patient well-being. Establishing this core outcome set through the Delphi method will provide a robust foundation for future clinical studies, fostering international consistency in outcome reporting and facilitating the development of targeted therapeutic interventions.

Conclusion

This Delphi-based study protocol aims to create a core outcome measure set for assessing recovery in patients with epilepsy and comorbid depression or anxiety, informed by the ICF framework. The consensus-driven approach will ensure that the outcome set captures essential aspects of recovery, supporting improved patient management and enhancing the quality of evidence in this complex population. Subsequent validation studies will be crucial to confirming the utility and applicability of this core set in diverse clinical settings.

.

Study Protocol

1.Study background

Anal Epilepsy is one of the most prevalent neurological disorders, affecting approximately 50 million people globally[1]. This condition is characterized by recurrent, unprovoked seizures resulting from abnormal electrical activity in the brain. Beyond the significant burden of seizure-related symptoms, many individuals with epilepsy experience various psychological and psychosocial challenges, further complicating their treatment and prognosis [2, 3]. Among these, comorbid mental health disorders—particularly depression and anxiety—are highly prevalent and contribute considerably to the overall disease burden[4]. It is estimated that up to 30-50% of patients with epilepsy also suffer from major depressive disorder or anxiety disorders, which are known to exacerbate the impact of epilepsy on patients' quality of life, social functioning, and treatment adherence [5, 6]. Such comorbid conditions often lead to poorer outcomes, increased healthcare utilization, and reduced efficacy of epilepsy treatments [7].

The impact of depression and anxiety on patients with epilepsy is profound and multifaceted. Depression, for example, has been associated with higher rates of suicidal ideation and suicide attempts in epilepsy populations [8, 9], highlighting the need for comprehensive, proactive mental health management in these patients. Anxiety disorders, on the other hand, can increase seizure frequency due to heightened physiological arousal, further impairing patients' ability to manage their condition effectively [10]. Moreover, comorbid mental health disorders are known to interfere with treatment adherence [11, 12], as patients with anxiety or depression may exhibit lower motivation to adhere to prescribed antiepileptic medications or lifestyle modifications. Despite the availability of various treatment modalities for epilepsy, including pharmacotherapy, neuromodulation, and behavioral interventions, the lack of a standardized, comprehensive tool to assess recovery outcomes remains a critical barrier in clinical practice. Without such tools, healthcare providers lack reliable methods to evaluate the full spectrum of post-treatment recovery in this complex population.

Current assessment approaches for post-treatment recovery in epilepsy often rely heavily on self-reported clinical symptoms and subjective evaluations by healthcare providers [13]. While these methods can provide insights into the patient's condition, they frequently lack standardization, objectivity, and reproducibility. Furthermore, traditional assessments tend to focus on seizure frequency and severity, often overlooking the broader psychosocial and functional dimensions of recovery that are especially relevant in patients with comorbid mental health conditions. For example, metrics that capture quality of life, emotional stability, cognitive function, and social reintegration are often underrepresented in conventional assessment tools, despite their importance in the holistic evaluation of patient recovery. As such, there is a pressing need for a more structured and standardized outcome measure set that reflects the complexity of recovery in epilepsy patients with comorbid depression or anxiety.

The development of a core outcome measure set offers a solution to these challenges by establishing standardized benchmarks that can be used across clinical trials and routine practice. The International Classification of Functioning, Disability, and Health (ICF) framework, developed by the World Health Organization, provides a comprehensive model for understanding health and disability [14, 15]. The ICF framework captures the interplay of biological, psychological, and social factors, making it an ideal foundation for developing a core outcome set that considers the multidimensional aspects of health [16, 17]. By mapping recovery outcomes onto ICF domains—such as physical health, mental well-being, and social participation—researchers can create a more holistic tool that is better suited to the needs of epilepsy patients with comorbid mental health disorders. The adoption of an ICF-based outcome set in epilepsy research could enhance the ability to monitor recovery across diverse patient populations and settings, ultimately leading to improved treatment strategies and better long-term outcomes [18, 19].

This study protocol outlines the development of an internationally agreed-upon core outcome measure set for evaluating post-treatment recovery in patients with epilepsy and co-occurring depression or anxiety disorders. To achieve this, we will employ a Delphi methodology, a structured, iterative process that enables

the systematic synthesis of expert opinion to reach consensus. The anticipated outcome of this study is to construct a validated, consensus-based core outcome set that will serve as a standardized assessment tool for evaluating post-treatment recovery in epilepsy patients with comorbid depression or anxiety. By addressing the physical, psychological, and social dimensions of health, this tool will enable clinicians and researchers to capture a comprehensive view of recovery that goes beyond seizure control. This standardized outcome set is expected to facilitate cross-study comparisons, support evidence synthesis in systematic reviews, and guide the development of personalized treatment approaches. Furthermore, it will provide a valuable resource for evaluating the efficacy of emerging therapies aimed at improving mental health and overall quality of life in epilepsy patients.

2. Part One: Design and Implementation of the Delphi Survey

Delphi Methodology for Developing Core Outcome Sets

This study employs the Delphi method to develop core outcome sets for the rehabilitation assessment of patients with epilepsy co-occurring with depression or anxiety. The choice of this method is based on its ability to systematically collect and synthesize expert opinions, making it particularly suitable for achieving broad consensus in medical and health domains.

Initial Preparation and Literature Review

The initial phase of the study focuses on an extensive literature review, aimed at collecting and assessing current literature related to the rehabilitation following treatment of epilepsy and its psychological complications. This includes systematically searching medical databases such as PubMed, Embase, and the Cochrane Library, using keywords such as “epilepsy,” “depression,” “anxiety,” “post-treatment rehabilitation,” and “outcome measurement.” Through this process, we are able to identify widely documented therapeutic effects and rehabilitation assessment indicators, providing a scientific basis for the subsequent Delphi survey design.

Literature selection and evaluation are conducted in accordance with the PRISMA (Preferred Reporting Items for Systematic Reviews and Meta-Analyses) guidelines [20], to ensure methodological transparency and replicability. The quality of evidence will be assessed using tools such as the Cochrane Risk of Bias tool for randomized controlled trials and the Newcastle-Ottawa Scale for observational studies [21, 22]. Through this phase, the team establishes a preliminary list of outcome indicators deemed crucial for assessing the therapeutic effects on epilepsy and its psychological complications.

Delphi Survey Design and Pilot Testing

After establishing a preliminary list of indicators, we design the Delphi survey questionnaire and first conduct a pilot test within a small group of experts. The pilot test primarily serves to verify the clarity of the questionnaire content and its effectiveness in measuring experts' views on various therapeutic effect indicators. Additionally, the pilot helps us optimize the questionnaire format and operational procedures to ensure smooth implementation across a broader group of experts.

Implementation of the Survey

The Delphi survey is conducted online using Google Forms, with participants including neurologists, psychiatrists, psychologists, and experienced patient representatives. We collect data anonymously to encourage participants to provide open and honest feedback. Each expert rates the outcome indicators using a 9-point Likert scale, where 1-3 points indicate the indicator is not important, 4-6 points suggest it is important but not critical, and 7-9 points indicate it is very important and must be included in the core outcome set.

Data protection measures, such as encryption technology for secure data transfer and storage, ensure participants' opinions remain anonymous. These data protection measures not only comply with ethical standards but also enhance the survey's effectiveness, as participants can express their true opinions freely while ensuring their personal information's security.

Sample Size

To achieve representative and effective consensus, the sample size for the Delphi panel is carefully calculated. Based on the guidelines for core outcome set development and prior empirical studies, the study initially recruits at least 20 participants from each stakeholder category, with an anticipated dropout rate of about 30%. This strategy ensures that each group still has sufficient representation (at least 14 participants) after attrition, to maintain the integrity and validity of the consensus results. Additionally, participants represent a broad geographic distribution and include various professional and experiential backgrounds, covering comprehensive insights on epilepsy treatment and rehabilitation processes.

Iterative Survey Process

In line with the standards of the Delphi method, we anticipate conducting at least three survey rounds to progressively refine and validate the outcome indicators. After each survey round, the research team conducts a detailed analysis of the collected data, calculating the average score and consistency index for each indicator. Based on these data, indicators may be modified or merged to maximize their relevance and importance.

Through this structured and iterative process, we expect to ultimately establish a scientifically rigorous and practical core outcome set. These indicators will not only be used to assess therapeutic effects but will also aid clinicians, researchers, and patients in better understanding and improving the treatment approaches for epilepsy and its psychological complications. Through the meticulous application of the Delphi method, this study aims to provide a solid and comprehensive foundation for the rehabilitation assessment of epilepsy with psychological disorders.

3. Data Management and Protection of Participants' Anonymity

In this study, employing the Delphi method to develop and validate rehabilitation assessment indicators for patients with epilepsy and concurrent depression or anxiety, data management and the

protection of participant anonymity are especially critical. This not only helps ensure the scientific integrity and objectivity of the survey but also represents a fundamental respect for participants' privacy rights.

Application of Data Anonymization and Encryption Technologies

To maximize the protection of participants' anonymity, this research has implemented stringent data anonymization measures from its inception. Each participant is assigned a randomly generated unique identifier at the start of the survey, which is completely detached from their personal identity information. All data collection, storage, and processing are identified using this code, ensuring the anonymity of personal information.

Moreover, to ensure the security of data transmission, the research team employs advanced encryption technologies. All online survey form transmissions are secured with Secure Sockets Layer (SSL) encryption, a widely used technology for protecting internet communications and sensitive data. SSL encryption not only secures the data during transmission but also prevents unauthorized access and data breaches.

Data Collection and Storage

Data collection is conducted through automated Google Forms, which is not only convenient and quick but also helps reduce data errors due to manual handling. Once data are entered, they are automatically saved on a password-protected server, accessible only to authorized members of the research team.

Access and use of data by the research team are also subject to strict regulations. Each team member must sign a confidentiality agreement before gaining access rights, specifying their responsibilities and obligations during the data handling process. Moreover, all operations involving data analysis must be conducted under the premise of ensuring data anonymity.

Integrity Checks and Monitoring of Data

To ensure the accuracy and completeness of the collected data, the research team has established a series of data audit processes. After each round of the Delphi survey, the data team conducts checks on data integrity, including verifying the completeness, consistency, and logic of the data. Any errors or inconsistencies found are immediately corrected to ensure the accuracy of the analyses.

This stringent data management mechanism not only protects participants' anonymity but also enhances the reliability of the survey data and the validity of the research results. Through these measures, the research team can be confident that the data accurately reflect the opinions and recommendations of experts, thereby providing scientific and effective assessment indicators for the treatment and rehabilitation of epilepsy and its psychological complications.

4. Extensive Stakeholder Involvement and Dissemination and Implementation of Results

In this study, we emphasize ensuring extensive stakeholder involvement and the effective dissemination and implementation of research findings. This not only increases the acceptability and practicality of the research outcomes but also helps translate these results into actual clinical practice improvements.

Identification and Involvement of Stakeholders

Firstly, identifying and incorporating various stakeholders is crucial to the success of this research. Our stakeholders include neurologists, psychiatrists, psychologists, rehabilitation specialists, patients, and their families. By identifying the specific needs and expectations of these groups, the research team can better design the survey questionnaire and ensure the results reflect a wide range of professional and patient perspectives.

To effectively incorporate these stakeholders, we employ multiple strategies. On one hand, we collaborate with professional associations to reach professionals through their networks and invite them to

participate in the Delphi survey. On the other hand, we also reach out to a broader group of patients and their families through patient support groups and social media platforms, ensuring their voices and needs are adequately considered.

Dissemination of Results

After completing the Delphi survey and forming the final set of core outcome indicators, the research team will proceed to prepare for the dissemination of results. This includes writing detailed research reports and papers, as well as presenting orally and through posters at professional conferences. The dissemination of these results aims to convey the methods, findings, and recommendations of the research to the medical community and the public, increasing the visibility and impact of the research.

We plan to publish research papers in open-access medical journals, ensuring that all stakeholders, whether medical professionals or patients, can access this critical information without barriers. Moreover, the research team will also utilize digital media, such as blogs and social media, to explain the research outcomes in more accessible language, thereby expanding its educational and advocacy roles.

Implementation of Results

After the research findings are published, the next crucial step is to implement these results, applying the core outcome indicator set developed by the research to clinical practice. To this end, we will collaborate with medical institutions and professional groups to develop training materials and guidelines to help doctors and rehabilitation specialists understand and adopt these new assessment tools.

Moreover, the research team plans to monitor the implementation effects of the indicator set, evaluating its effectiveness and feasibility in real medical settings through regular feedback and implementation data collection. This information will be used to continuously optimize and adjust the indicator set, ensuring it truly improves the rehabilitation processes for patients with epilepsy and its psychological complications.

Through this comprehensive and in-depth approach, this research not only provides a set of scientifically validated rehabilitation assessment tools but also promotes effective participation and deep collaboration among a wide range of stakeholders, thereby driving continual progress and innovation in the treatment field of epilepsy and its psychological complications.

References

1. Vaughan, K.A., et al., An estimation of global volume of surgically treatable epilepsy based on a systematic review and meta-analysis of epilepsy. J Neurosurg, 2019. 130(4): p. 1127-1141.
2. Keikelame, M.J., et al., Psychosocial challenges affecting the quality of life in adults with epilepsy and their carers in Africa: A review of published evidence between 1994 and 2014. Afr J Prim Health Care Fam Med, 2017. 9(1): p. e1-e5.
3. Mula, M. and J.W. Sander, Psychosocial aspects of epilepsy: a wider approach. BJPsych Open, 2016. 2(4): p. 270-274.
4. Keezer, M.R., S.M. Sisodiya, and J.W. Sander, Comorbidities of epilepsy: current concepts and future perspectives. Lancet Neurol, 2016. 15(1): p. 106-15.
5. Lu, E., et al., Systematic Literature Review of Psychiatric Comorbidities in Adults with Epilepsy. J Clin Neurol, 2021. 17(2): p. 176-186.
6. Pham, T., et al., The prevalence of anxiety and associated factors in persons with epilepsy. Epilepsia, 2017. 58(8): p. e107-e110.
7. Patel, R.S., et al., Psychiatric Comorbidities and Outcomes in Epilepsy Patients: An Insight from a Nationwide Inpatient Analysis in the United States. Cureus, 2017. 9(9): p. e1686.
8. Friedman, D., et al., Depressive symptoms and suicidality among individuals with epilepsy enrolled in self-management studies: Results from the US Centers for Disease Control and Prevention Managing Epilepsy Well (MEW) Network. Epilepsy Behav, 2018. 87: p. 235-240.
9. Nigussie, K., et al., Magnitude and associated factors of suicidal ideation and attempt among people with epilepsy attending outpatient treatment at primary public hospitals in northwest Ethiopia: a multicentre cross-sectional study. BMJ Open, 2021. 11(1): p. e043227.
10. Dehn, L.B., et al., Relationships of depression and anxiety symptoms with seizure frequency: Results from a multicenter follow-up study. Seizure, 2017. 53: p. 103-109.
11. Roca, M., et al., Adherence to antidepressant treatment in depressive patients with comorbid psychiatric disorders. European Psychiatry, 2011. 26(S2): p. 1277-1277.
12. Litz, M. and D. Leslie, The impact of mental health comorbidities on adherence to buprenorphine: A claims based analysis. Am J Addict, 2017. 26(8): p. 859-863.
13. Mücke, F.J., et al., Discrepancy between subjective and objective memory change after epilepsy surgery: Relation with seizure outcome and depressive symptoms. Front Neurol, 2022. 13: p. 855664.
14. Huang, Y., et al., Application of the International Classification of Functioning, Disability and Health (ICF) in dementia research and practice: A scoping review. Aging Ment Health, 2023. 27(2): p. 357-371.
15. Leonardi, M., et al., 20 Years of ICF-International Classification of Functioning, Disability and Health: Uses and Applications around the World. Int J Environ Res Public Health, 2022. 19(18).
16. Bornbaum, C.C., et al., A critical exploration of the International Classification of Functioning, Disability, and Health (ICF) framework from the perspective of oncology: recommendations for revision. J Multidiscip Healthc, 2013. 6: p. 75-86.
17. Ewert, T., et al., Validation of the International Classification of Functioning Disability and Health framework using multidimensional item response modeling. Disabil Rehabil, 2010. 32(17): p. 1397-405.
18. Crudgington, H., et al., Core Health Outcomes in Childhood Epilepsy (CHOICE): Development of a core outcome set using systematic review methods and a Delphi survey consensus. Epilepsia, 2019. 60(5): p. 857-871.

19. Noble, A.J. and A.G. Marson, Which outcomes should we measure in adult epilepsy trials? The views of people with epilepsy and informal carers. Epilepsy Behav, 2016. 59: p. 105-10.

20. Page, M.J., et al., PRISMA 2020 explanation and elaboration: updated guidance and exemplars for reporting systematic reviews. Bmj, 2021. 372: p. n160.

21. Higgins, J.P., et al., The Cochrane Collaboration's tool for assessing risk of bias in randomised trials. Bmj, 2011. 343: p. d5928.

22. Stang, A., Critical evaluation of the Newcastle-Ottawa scale for the assessment of the quality of nonrandomized studies in meta-analyses. Eur J Epidemiol, 2010. 25(9): p. 603-5.23.
