# Supplementary material for: Development of a consensus-based core outcome set for post-treatment recovery in adults with epilepsy and comorbid depression or anxiety: A Delphi and ICF-guided protocol
Source: PLoS One. 2025 Aug 22;20(8):e0330617. doi: 10.1371/journal.pone.0330617 (PMC12373203; doi:10.1371/journal.pone.0330617)
Supplement: S3 File — (DOCX) [file pone.0330617.s003.docx]

**Supplementary Material**

**Search Strategery for Pubmed**

#1 "Epilepsy"[Mesh] OR "Seizures"[Mesh]

#2 ("epilepsy"[Title/Abstract] OR "seizure"[Title/Abstract])

#3 #1 OR #2

#4 "Depression"[Mesh] OR "Depressive Disorder"[Mesh]

#5 "depression"[Title/Abstract] OR "depressive disorder"[Title/Abstract]

#6 #4 OR #5

#7 "Anxiety"[Mesh] OR "Anxiety Disorders"[Mesh]

#8 "anxiety"[Title/Abstract] OR "anxiety disorders"[Title/Abstract]

#9 #7 OR #8

#10 "Recovery of Function"[Mesh] OR "Treatment Outcome"[Mesh] OR "Patient Discharge"[Mesh] OR "Follow-Up Studies"[Mesh]

#11 "recovery"[Title/Abstract] OR "outcome"[Title/Abstract] OR "follow-up"[Title/Abstract]

#12 #10 OR #11

#13 #6 OR #9

#14 #3 AND #12 AND #13
